# Ecology of urban malaria vectors in Niamey, Republic of Niger

**DOI:** 10.1186/s12936-016-1352-0

**Published:** 2016-06-08

**Authors:** Rabiou Labbo, Thierry Fandeur, Isabelle Jeanne, Cyril Czeher, Earle Williams, Ibrahim Arzika, Amadou Soumana, Ramatoulaye Lazoumar, Jean-Bernard Duchemin

**Affiliations:** Centre de Recherche Médicale et Sanitaire (CERMES), Institut Pasteur International Network, 634 Bd de la Nation, BP 10887, Niamey, Niger; School of Medicine, Deakin University, 75 Pigdons Road, Waurn Ponds, VIC 3216 Australia; World Health Organization Representative Offices for Solomon Islands, Honiara, Solomon Islands; Parsons Laboratory, Massachusetts Institute of Technology (MIT), Cambridge, MA USA; Hôpital National de Niamey, Niamey, Niger; Australian Animal Health Laboratory (AAHL), Health and Biosecurity CSIRO, 5 Portarlington Road East, Geelong, VIC 3220 Australia

**Keywords:** Malaria, Niamey, Anopheles, *Anopheles gambiae*, Vector ecology, Urban, Sahel, Niger

## Abstract

**Background:**

Urbanization in African cities has major impact on malaria risk. Niamey, the capital of the Republic of Niger, is situated in the West African Sahel zone. The short rainy season and human activities linked with the Niger River influence mosquito abundance. This study aimed at deciphering the factors of distribution of urban malaria vectors in Niamey.

**Methods:**

The distribution of mosquito aquatic stages was investigated monthly from December 2002 to November 2003, at up to 84 breeding sites, throughout Niamey. An exploratory analysis of association between mosquito abundance and environmental factors was performed by a Principal Component Analysis and confirmed by Kruskall–Wallis non-parametric test. To assess the relative importance of significant factors, models were built for *Anopheles* and Culicinae. In a second capture session, adult mosquitoes were collected weekly with pyrethrum sprays and CDC light-traps from June 2008 to June 2009 in two differentiated urban areas chosen after the study’s first step. Members of the *Anopheles gambiae* complex were genotyped and *Anopheles* females were tested for the presence of *Plasmodium falciparum* circumsporozoite antigens using ELISA.

**Results:**

In 2003, 29 % of 8420 mosquitoes collected as aquatic stages were *Anopheles*. They were significantly more likely to be found upstream, relatively close to the river and highly productive in ponds. These factors remained significant in regression and generalized linear models. The Culicinae were found significantly more likely close to the river, and in the main temporary affluent stream. In 2009, *Anopheles* specimens, including *Anopheles gambiae s.l.* (95 %), but also *Anopheles funestus* (0.6 %) accounted for 18 % of the adult mosquito fauna, with a large difference between the two sampled zones. Three members of the *An. gambiae* complex were found: *Anopheles arabiensis*, *Anopheles coluzzii*, and *An. gambiae*. Nineteen (1.3 %) out of 1467 females tested for *P. falciparum* antigen were found positive.

**Conclusion:**

The study provides valuable update knowledge on malaria vector ecology and distribution in Niamey. The identification of spatial and environmental risk factors could pave the way to larval source management strategy and allow malaria vector control to focus on key zones for the benefit of the community.

**Electronic supplementary material:**

The online version of this article (doi:10.1186/s12936-016-1352-0) contains supplementary material, which is available to authorized users.

## Background

There is a crucial need for a better understanding of the malaria vector ecology in Niamey and quantification of transmission. Knowledge about malaria transmission and vector ecology in this capital city is outdated and may be insufficient to implement adapted vector control measures. Urban human populations increase rapidly, especially in West Africa. In this region, the population growth is twice as much the general population growth [1]. This has major implications for malaria risk and control [2]. The epidemiology of malaria in African urban areas is characterized by a declining gradient of transmission from the periphery to the centre [2, 3]. This is due to the limited flight capacity and spread of *Anopheles* in urban environment and to the scarcity and pollution level of potential breeding sites. In urban environments, lower densities of larval habitats are found relative to rural environments [2, 4]. This presumably fosters greater interactions, including competition, between the different taxa of *Anopheles gambiae* sensu lato and other mosquito species, often in a context of polluted water bodies.

Niamey is located within the Sahel zone. The population is about two million people. The rainy season is short and intense from June to October. Locally, people use to manage their water needs in coherence with the scarcity of water during the dry season and, for some of them, to adopt agricultural and farming practices in a sustainable manner. Historically, Niamey was settled on the left bank of the Niger River, but now it extends largely on both banks and a large portion of the city is notably distant from the river. The periphery of the city reaches and integrates several rural villages. The Niger River has allowed the development of irrigation schemes for rice cultivation and many rice fields are at short distance from the city centre. Beside these modern practices, traditional agricultural activities as gardening for fruits, vegetables and flowers are carried out in town. Malaria in rural Sahel is strongly seasonal [5, 6]. It peaks during and shortly after the rainy season. However transmission could occur at very low level during the dry seasons. In specific edaphic conditions, where the water table is close to the surface or due to permanent or semi-permanent water bodies, breeding sites for malaria vectors rely less upon rainfall and could last well beyond the rainy season and induce a longer transmission period [7]. Entomological data about malaria vectors in Niamey come from studies [8, 9] led before the drought period beginning 40 years ago. The authors described *Anopheles gambiae s.l.* and *Anopheles funestus* as the major potential vectors to be present. In 1973, Chauvet and Dyemkouma [8] focused their attention on the *kanaris*, the domestic adobe vases used for daily water storage and did not find any *Anopheles* in them. In 1997, Julvez et al. [3] published prevalence data on malaria parasites in humans and defined the period of malaria transmission in Niamey as the 5 months of the rainy season (June to October), with more intense transmission in neighborhoods close to the Niger River and less intense transmission in districts further away the river, differing to the classical schema of lower level of malaria transmission in urban centres [2]. Such a situation has already been described in Libreville, Gabon [10]. However the authors emphasize the role of socio-cultural factors rather than environmental ones. In a wide range of transmission magnitude, and considering this relative low density of breeding sites available for malaria vectors and the high density of human population susceptible to malaria infections, urban environments could present a high benefit-cost ratio for vector control, and especially larval control [11]. In comparison, rural zones could offer a wider range of breeding sites for malaria vectors, dispersed on larger areas, making the vector control more critical. The human population is less dense than in urban areas and a bigger effort, in logistics and funding, is needed to protect a comparable population. The World Health Organization (WHO) maintains its strong advice for use of long-lasting insecticidal nets (LLINs) and indoor residual spraying (IRS) as the backbone for malaria vector control. However, the WHO issued in 2012 a position statement on larviciding [12]. The method was recommended as a supplement in situations “where vectors tend to breed in permanent or semi-permanent water bodies that can be readily identified and accessed, i.e. breeding sites which are ‘few, fixed and findable’, and where the density of the human population to be protected is sufficiently high to justify the necessary resources” [12]. The experience of Dar-es-Salaam, Tanzania, in term of larval control [13] has proven it to be cost-effective in urban area [11]. The Niger has implemented a nation-wide impregnated bed net distribution [6]. Time has come to reinforce the malaria control. The larval source management, including larviciding method, could be combined to LLINs and IRS to decrease the malaria burden.

The study aims at characterizing the ecology and the distribution of pre-imago stages of *Anopheles* vectors in view of the potential importance of the Niger River and new agricultural practices. In a second step the study confirms the pre-imago ecology results with quantification of adult mosquito abundance and infection in two differentiated zones of Niamey.

## Methods

### Study site

**Fig. 1:**
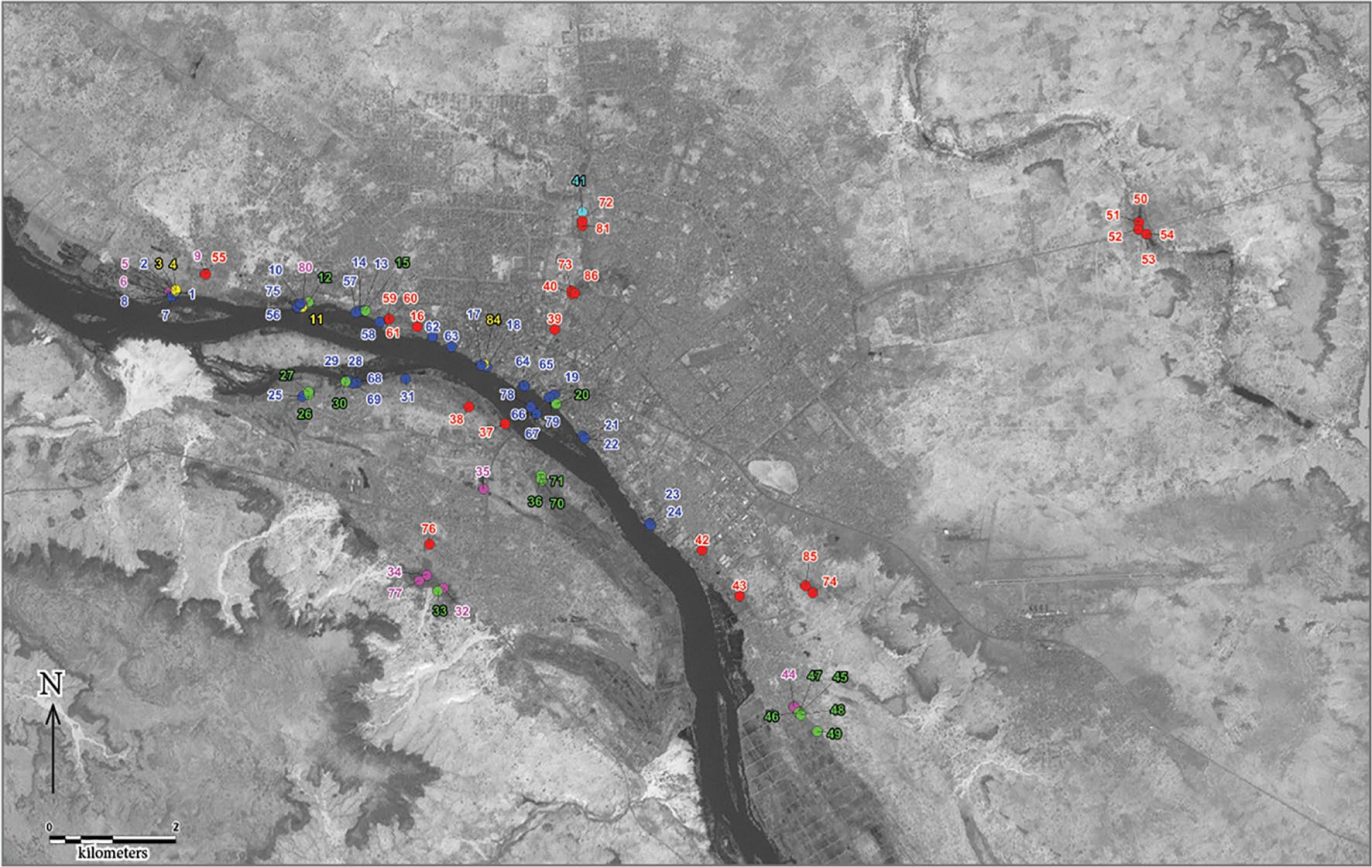
Distribution of the 84 studied larval sites. The sites have been grouped into six main classes according to their typology (see text): Human made (*red*), Ricefield (*green*), Niger River (*blue*), Kori (*light blue*), Pond (*pink*) and very small sites (*yellow*). The background is extracted from a satellite image of LANDSAT ETM+ band 8, acquired on 2nd December 1999. The river is at a high water level

The area is located in Niamey (Fig. 1) in the southwestern region of Niger, Africa, (13°31′N, 2°06′E). Niamey has a semi-arid climate, with a rainy season lasting about 5 months (June to October) during which 99 % of annual rainfalls occur. The rainy season peaks in August, but local dry spells of up to 10 days are possible even during this month, the temporal distribution of rainfall being extremely irregular [14]. The coolest months are November to February, with the Harmattan (an arid trade wind particularly prevalent from December to February). The mean monthly maximum temperature reaches 40 °C in April, during the dry and hot season from March to May, and a low mark of 19 °C in December. The Niger River is the principal permanent body of water in the city, but there are widespread hollows that are transformed into temporary pools during the rainy season, some of which may persist during the cold and hot periods of the dry season, thereby constituting potential breeding sites for mosquitoes. The conurbation extends over an area of more than 255 km^2^, still unevenly distributed on either side of the Niger River (Fig. 1).

### Aquatic stages collection sites

**Fig. 2:**
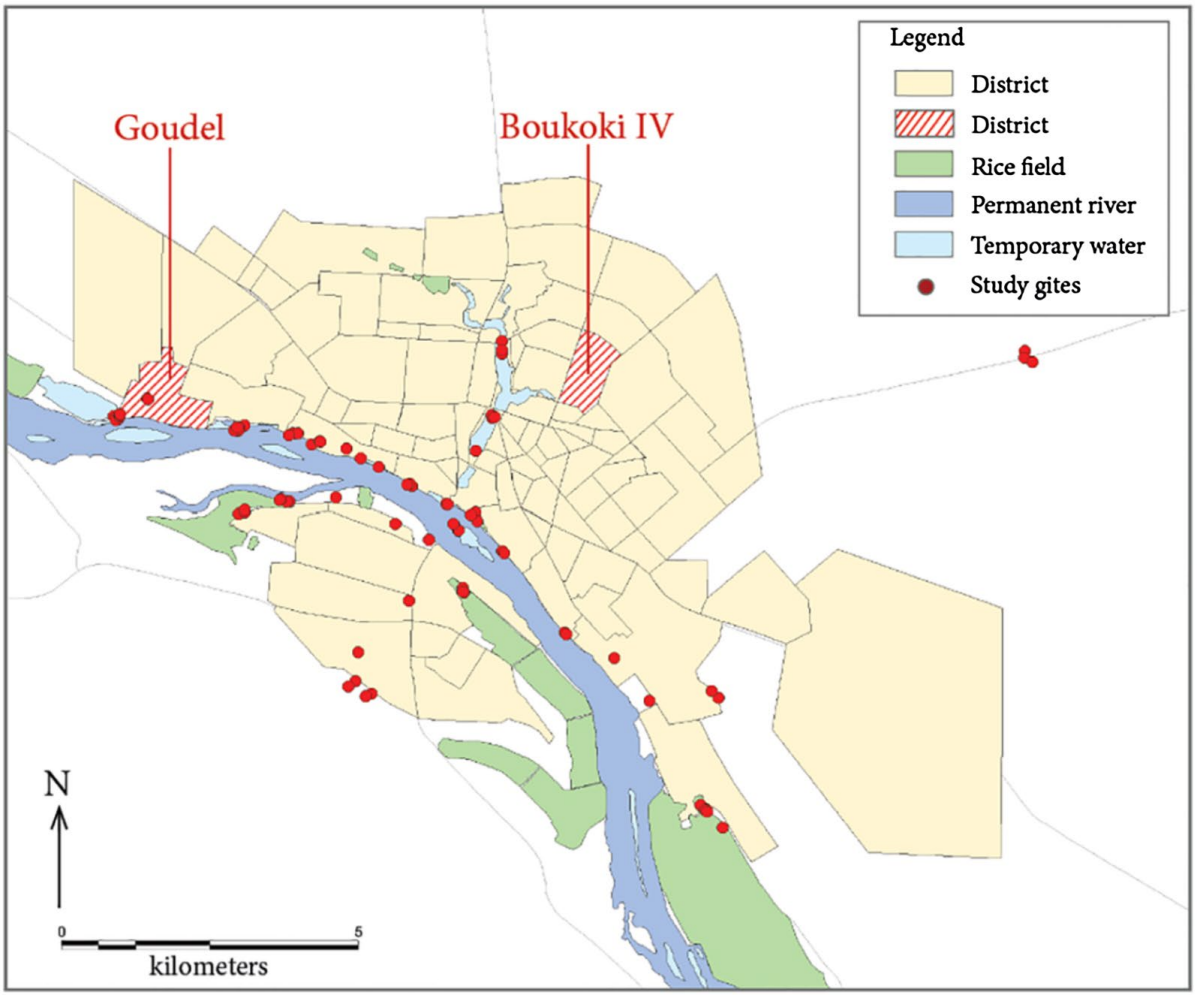
Situation of the two districts where mosquito adults were collected. Situation of the two districts where mosquito adults were collected in 2009, in red-striped surfaces. *Red circles* indicate the study sites for aquatic stages. The administrative limits of urban districts are in *pale yellow*; the main Niger Riverbed is *deep blue*; significantly large but temporary water bodies (Kori, flooded zones) are *pale blue*; rice fields are *green*

The survey first focused on the vicinity of the Niger River and its affluent the Gountou Yena kori, which crosses the town, but many other sites were sampled: ponds, drains, gardens, flooded areas, puddles, irrigation systems, with channels and wells. For a better framing of the rainy season, the number of sampled sites increased from 33 at starting in November 2002, to a total of 84 water bodies until the end of the larval and pupae survey period (Figs. 1, 2).

### Aquatic stages collection and specimen processing

Larvae and pupae were sampled by a standard dipping method: a total of 10 dips per aquatic habitat were carried out, or less if the site water volume was not enough. The total of the larvae and pupae collected with these dips were quickly sorted on site in a white tray according to the sub-family level by eye as *Anopheles*, the only genus of the subfamily in the zoneoras Culicinae. Then they were counted according to their stage of development: first, second, third or fourth instars and grouped together in breeding containers for each positive site. The larvae were kept and fed with finely ground dog food until the adult stage. Once emerged, the adults were identified morphologically under a stereomicroscope, as described below. A subset of *An.gambiae s.l.* specimens were genotyped (see below).

### Geographic information system

Geographical coordinates of study sites were collected with a GPS device (Garmin12) and recorded in a Geographic Information System built with MapInfo Professional (Version 8.5.1 © 2006 MapInfo Corporation) and Quantum GIS (version 1.7.4-Worclaw) [15]. The sites were characterized by their orthogonal distance to the main stream (median of the riverbed line), ordered as following: “1”, <500 m, “2”, 500–1500 m, “3” >1500 m; and by their relative situation along the river stream, beginning by the most upstream site at 0 m: upstream (<2500 m, “1”) or downstream (>6000 m, “3”) or at the city level (2500–6000 m, “2”). The dataset was built up with mosquito aquatic stages and absolute numbers for *Anopheles* and Culicinae by site and by month. The breeding sites were characterized by their typology and the estimated surface of the water body. The typology of each breeding sites was described in six modalities: ‘river’, ‘pond’, ‘human made’ gathering wells, channels and drains, ‘rice field’ and ‘small breeding sites’ (recipients, footprints) and ‘kori’. The total surface of the sampled site was assessed by eye, as a whole and coded as follows: “0” if dry, “1” <0.1 m^2^, “2” <1 m^2^, “3” <10 m^2^, “4” <100 m^2^, “5” <1000 m^2^, “6” ≥1,000 m^2^, “7” in relation with the river bed and “8” if no access was available. The sampling and coding were performed by a team composed of several persons who shared their estimations.

### Adult mosquito collection and specimen processing

From June 2008 to May 2009, a longitudinal entomological survey was carried out in two districts, selected on the basis of the results of the 2003 study: (1) Boukoki, in the city centre and distant to the Niger River and (2) Goudel, a semi-urban district relatively close to the River Niger, upstream from the city center and next to a pond (Fig. 2).

In each study site, both traditional and modern houses were randomly selected and sampled for mosquito collection, after agreement of householders.

- Adult mosquitoes were collected weekly by two sampling methods:
- Indoor pyrethrum spray catches: two houses (one room per house) per district were sprayed with insecticide early in the morning (between 6:00 and 9:00 am) for the collection of the resting female mosquitoes. Knocked-down female mosquitoes were quickly collected from white sheets laid on the floor and the furniture of sprayed rooms.
- Indoor CDC light-traps (CDC miniature light trap, Model 512, John W. Hock Co., Gainesville, FL): Three other houses (one room per house) close to the indoor pyrethrum sprayed houses were sampled. Light traps were set up at 7:00 pm and mosquitoes were collected at 7:00 am.

The mosquitoes were identified at the genus or species level for *Anopheles* on the basis of morphological criteria, with the identification keys of Gillies and De Meillon [16] and Gillies and Coetzee [17]. Each *Anopheles* specimen was stored in an individual well of a 96-well plate and stored at −20 °C until processing for molecular genotyping and ELISA-CSP testing.

### PCR identification of the *Anopheles gambiae* complex

For the two sampling periods, subsets of the *An. gambiae* complex individuals were identified by the polymerase chain reaction (PCR), as described by Scott et al. [18] targeting *An. gambiae s.s,* and *Anopheles arabiensis*. Further, *An. gambiae* specimens were identified on the basis of their ribosomal RNA genes as previously belonging to the Mopti/M molecular form or Savannah/S molecular form, as described by Favia et al. [19] and Santolamazza et al. [20]. However, due to the recent erection of the M form and the Mopti chromosomal form to *Anopheles coluzzii* [21] and the nomination of the S form and the Savannah chromosomal form as *An. gambiae*, results are expressed referring to this new taxonomic revision.

### ELISA for the detection of parasite infections in *Anopheles gambiae s.l.*

Specimens of *Anopheles* caught as adults during the second study were tested for the presence of the *Plasmodium falciparum* circumsporozoite protein (CSP) in the head and the anterior third of thorax. ELISA was carried out as described by Burkot et al. [22] and Wirtz et al. [23]. The heads of uninfected laboratory-reared mosquitoes were used as negative controls and a 10 μg/ml CSP antigen solution was used as a positive control. Samples were considered positive if absorbance values were more than twice the mean value for the four negative controls. Sporozoite indices (i.e. salivary gland infection rates) were estimated from the ratio of positive specimens on head/thorax on tested specimens.

### Analysis and statistics of the aquatic stage ecology

In a first step, exploratory analysis of the association between abundances of mosquito aquatic stages and environmental factors was performed with the ade4 [24] package for R-CRAN. A principal component analysis (PCA) was drawn out for both Culicinae and *Anopheles* followed by a between-groups analysis with environmental factors [25]. The statistical significance was assessed with permutation tests (Monte-Carlo procedure with 1000 permutations). Then bilateral associations were tested by a Kruskall–Wallis non-parametric test with the Rcmdr [26] package on each of the Culicinae and *Anopheles* datasets for each variable. Two variables were temporal: Season (Rainy: June to October, Cold Dry: November to February and Hot Dry: March to May), and Month of collection (=12). The variable Sites including 84 modalities, was stored as information as supplementary variable in the dataset and used in the PCA but not for model analysis. In addition to the variables describing the typology and the surface of the water bodies, two other variables addressed the spatial dimension of the data set, both with three ordered modalities: the distance to the river bed DistFlFAC (<500 m, 500–1500 m, and >1500 m) and the situation upstream or downstream to the city UpDown (‘upstream’, ‘city’, and ‘downstream’). To assess the hierarchy of the effects of the variables and to estimate the interaction between variables, two models were built: logistic regression (LG) with binomial distribution and the response variable (mosquito) set as presence/absence (0/1), and generalized linear model (GLM) with Poisson distribution for the response variable set as counts. Interaction between variables was tested by the effect of the ‘Season’ on the typology of sites and the distance to the river. The data included high number of zeros (dry breeding sites, no access). To minimize this effect, a dataset excluding the sites which were either dry or not accessible (coded ‘0’ or ‘8’ in the surface size variable) was built up and used in modeling. The goodness of model was visually assessed by the distribution of the residuals which should follow a Gaussian or near-Gaussian distribution. The residual deviance was compared to the null deviance. The best model within each model family was chosen after the Akaike Information Criterion (AIC).

## Results

### Aquatic stages survey

A total of 5223 dips collected 8420 larva and pupae. Among them, 2434 *Anopheles* (29 %) and 5986 Culicinae (71 %) were counted. Among *Anopheles* aquatic stages, *An. gambiae s.l.* represented the major part (96 %), *Anopheles rufipes*, *Anopheles pharoensis* and *Anopheles ziemanni* being present in small numbers. The Culicinae mosquito aquatic stages were identified as *Culex* sp. (90 %), *Mansonia* sp. (6 %) and *Aedes* sp. (4 %). Eight species of *Culex* were identified, and *Culex pipiens group* (*Culex quinquefasciatus*), and *Culex antennatus* predominated.

### Ecological study: anopheles ecological preferences

**Fig. 3:**
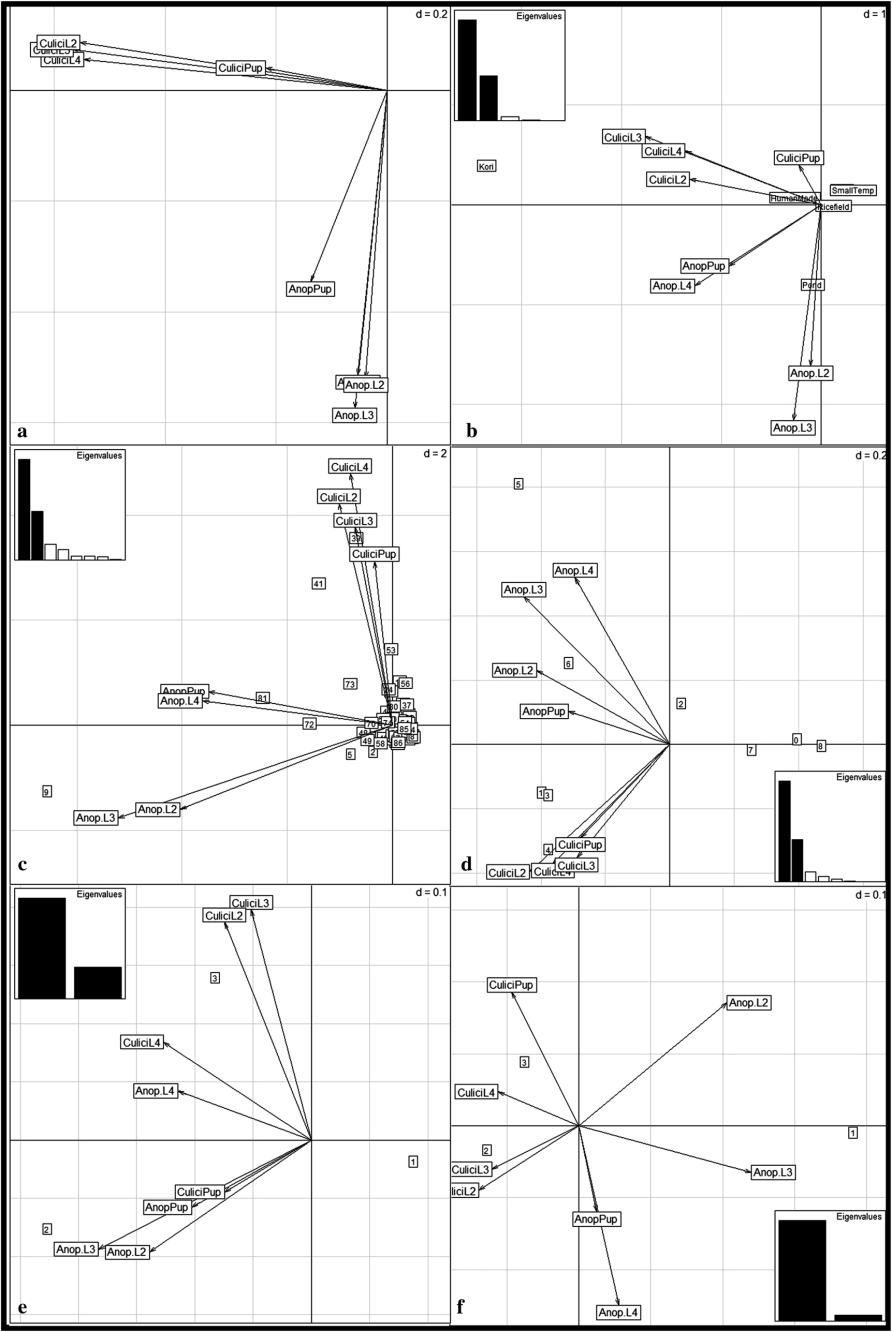
Principal component analysis (PCA) and between classes analysis (BCA) of *Anopheles* and Culicinae aquatic stages. ‘CuliciL2-3-4’ means Culicinae larval stage 2-3-4, ‘CuliciPup’ = Culicinae Pupa, ‘Anop.L2-3-4’ = *Anopheles* Larval stage 2-3-4, ‘AnopPup’ = *Anopheles* pupae. **a** Principal component Analysis. The *horizontal axis* is mainly defined by the Culicinae stages and the *vertical axis* by *Anopheles* stages. **b** BCA according to the typology of sampled sites. The ‘River’ tag is hidden behind the ‘Small Temp’ tag. The ‘Pond’ tag is within the space of *Anopheles* vectors. The ‘Kori’ tag aligns with the Culicinae vectors. **c** BCA according to the sites, the site ‘9’ is the Goudel Pond; the 41 site is the Kori (Fig. 1). **d** BCA according to the surface of water bodies. The three values ‘0’, ‘7’ and ‘8’ correspond respectively to sites which are dry, or in relation to the Niger River (>10,000 m^2^), or not accessible. They are at the opposite of the mosquito space of vector projection. **e** BCA according to the distance to the NigerRiver. ‘1’ at <500 m to the median line of the river bed, ‘2’ between 500 and 1500 m and ‘3’ beyond 1500 m from the river median line, meaning in the city centre. **f** BCA according to the up or down stream situation of sampled sites; ‘1’ is upstream, ‘2’ is at the city level, ‘3’ is downstream the city. Please note that the ‘*Anopheles*’ vectors all expand within the right part of the space (~X-axis, given the very high Eigenvalue of this axis, as shown in the bottom right legend)

The PCA performed on the *Anopheles* and Culicinae datasets with the three last larval and pupal stages by collection site and by month showed a different distribution of the two groups of taxa according to two main axes (Fig. 3a). These two first axes held for 61 % of the total variance of the data. They were comparable (respective Eigen values of 2.61 and 2.26) and supported the variance of Culicinae (x-axis) and *Anopheles* specimens (y-axis) (Fig. 3a).

To assess the effect of each environmental factor on the mosquito distribution, the ‘between-classes analysis’ option was performed on this PCA against the variables: ‘typology’ (Fig. 3b), ‘site’ (Fig. 3c), ‘season’, ‘surface’ (Fig. 3d) of breeding sites, the orthogonal distance to the Niger River ‘distf’ (Fig. 3e) and the up-downstream situation (Fig. 3f). All these associations, tested by a Monte Carlo procedure with 1000 permutations, gave significant values except for the ‘season’ factor (p = 0.194). More specifically, according to the typology of breeding sites (p = 0.002), the *Anopheles* stages seemed more associated with the ‘Pond’ value when the Culicinae tend to be linked to the ‘Kori’ and ‘Human made’ breeding sites (Fig. 3b). If the analysis targeted the sites themselves, the *Anopheles* showed a strong association (p = 0.001) with ponds or wells in gardens (Figs. 1, 3c the sites 9, 72, 81 and 5) when the Culicinae are linked to the kori (Figs. 1, 3c the sites 39, 41), and to wells in gardens (Figs. 1, 3c the sites 53 and 73), but different from those associated with the *Anopheles*. If the surface of the water bodies is tested (p = 0.004), the mosquito vectors were on the opposite side to the values 0 (dry), 8 (no access) and 7 (Niger River). *Anopheles* were more likely to be found in larger water bodies (5 and 6) than the Culicinae (1, 3 and 4) (Fig. 3d). The negative effect of the river was confirmed if the distance to the Niger River is assessed (Fig. 3e) and *Anopheles* were more often found between 500 and 1500 m to the river (like the Goudel pond). The Culicinae tended to be more abundant distantly from the river (p = 0.001). The situation of the sites along the stream showed (p = 0.004) that the *Anopheles* tend to breed in sites situated upstream and the Culicinae at the level of, or downstream of the city (Fig. 3f).

### Ecological study: non parametric test

**Fig. 4:**
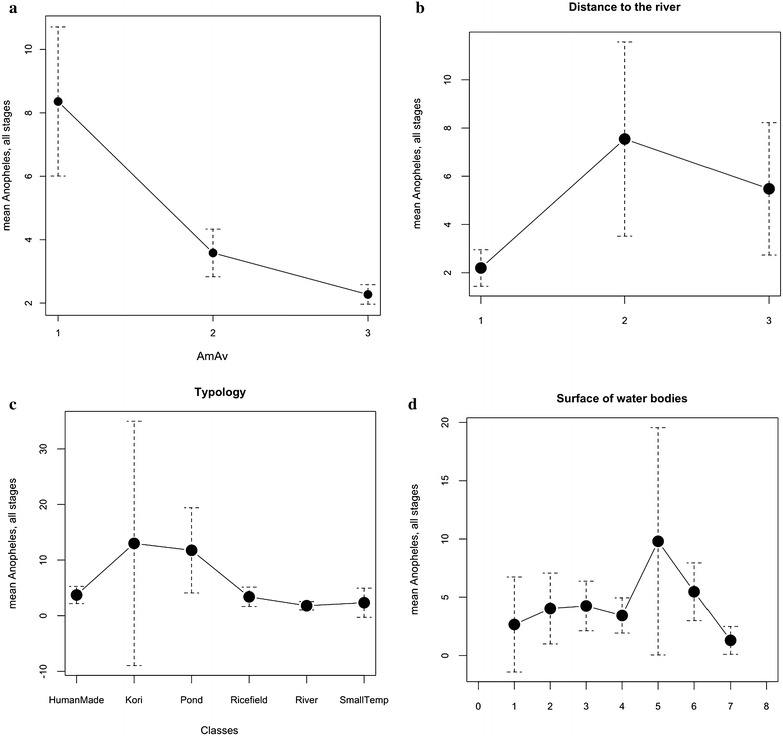
Means of *Anopheles* aquatic stages according to environment factors. **a** Upstream (‘1’), at the city level (‘2’) and downstream (‘3’). **b** Distance to the river: <500 m from the river median line (‘1’), between 500 and 1500 m (‘2’) and beyond 1500 m (‘3’). **c** Typology of sampled breeding sites. **d**: According to the surface of water bodies equals decimal log from <0.1 m^2^ (=1) to <1000 m^2^ (=5), >1000 m^3^ (=6), in contact with the river (=7). The ‘8’ (no access) or ‘0’ (dry) values have not been sampled

As the mosquito data are not normally distributed, the Kruskall–Wallis non-parametric test was used to test the associations between environmental factors and mosquito data. Significant associations were confirmed between *Anopheles* and the upstream/downstream situation (p = 0.011) with higher values upstream (Fig. 4a; Table 1). *Anopheles* were less abundant close to the river with the distance to the Niger River factor (p < 0.0001) (Fig. 4b; Table 2). The ‘Kori and ‘Pond’ classes of typology (Fig. 4c; Table 3) presented higher values (p = 0.0005) of *Anopheles* abundances. The larger water bodies (p < 0.0001) were found with more *Anopheles* (Fig. 4d). The relationship between *Anopheles* and season remained not significant (p = 0.077) (Table 4).

**Table 1:** Upstream to downstream

	*Anopheles*			Culicinae		
	n	Mean	SD	n	Mean	SD
Upstream	936	8.43	24.99	423	3.81	15.93
City	971	3.71	12.54	3723	14.21	62.60
Downstream	527	2.25	4.68	1837	7.85	23.16

*Anopheles* and Culicinae aquatic stages collected according to the situation of sites along the river stream. Data show mean, standard deviation and number of collection events (one per site and sampling time)

**Table 2:** Distance to the river

	*Anopheles*			Culicinae		
	n	Mean	SD	n	Mean	SD
<500 m	802	2.247	7.429	2281	6.389	23.942
500–1500 m	958	7.484	22.853	1591	12.430	53.171
>1500 m	674	5.525	15.427	2111	17.303	71.041

*Anopheles* and Culicinae aquatic stages collected according to the situation of sites from the riverbed. Data show mean, standard deviation and number of collection events (one per site and sampling time)

**Table 3:** Site classes

Site class	*Anopheles*			Culicinae		
	n	Mean	SD	n	Mean	SD
Human made	711	3.76	10.96	3030	16.03	49.71
Kori	130	13.00	30.71	786	78.60	234.22
Pond	776	11.76	31.16	233	3.53	13.59
Ricefield	400	3.36	9.48	692	5.82	15.80
River	396	1.85	5.72	1196	5.59	23.93
Small temperature	21	2.33	3.43	46	5.11	9.69

*Anopheles* and Culicinae aquatic stages collected by typology classes. Data show mean, standard deviation and number of collection events (one per site and sampling time)

**Table 4:** Seasons

	*Anopheles*			Culicinae		
	n	Mean	SD	n	Mean	SD
Dry cool	737	3.96	15.69	2736	14.71	71.64
Dry hot	590	3.99	13.34	1121	7.57	24.98
Rainy	1107	4.06	13.01	2126	7.79	22.54

*Anopheles* and Culicinae aquatic stages collected by season. Data show mean, standard deviation and number of collection events (one per site and sampling time)

**Fig. 5:**
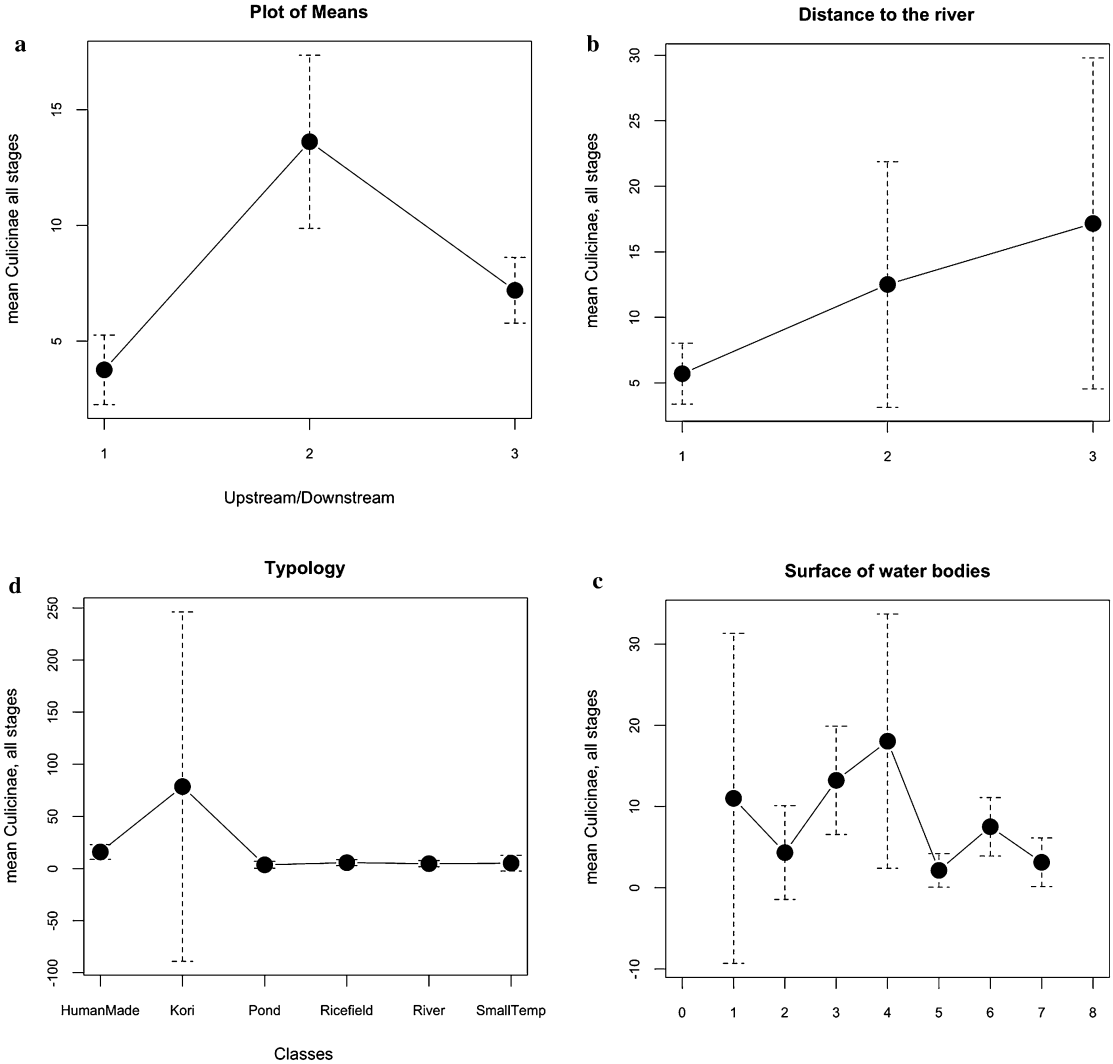
Means of Culicinae aquatic stages according to environment factors. **a** Upstream (‘1’), at the city level (‘2’) and downstream (‘3’). **b** Distance to the river: <500 m from the river median line (‘1’), between 500 and 1500 m (‘2’) and beyond 1500 m (‘3’). **c** Typology of sampled breeding sites. **d** According to the surface of water bodies equals decimal log from 0.1 m^2^ (=1) to <1000 m^2^ (=5), >1000 m^2^ (=6), in contact with the river (=7). The ‘8’ (no access) or ‘0’ (dry) values have not been sampled

For Culicinae, significant associations were still confirmed for the distance to the Niger River (p < 0.0001) with more Culicinae when the distance to the river increases (Fig. 5b; Table 2), the typology (p < 0.0001) with higher values with the ‘Kori’ and, to a lesser degree with ‘Human made’ breeding sites (Fig. 5c; Table 3), and the water surface (p < 0.0001) with a wide range of size of the water bodies (Fig. 5d), but smaller than the *Anopheles* (Fig. 3). The relationship with the situation along the stream (up/down) (Fig. 5a; Table 1) was not confirmed (p = 0.056), neither the seasonal effect (p = 0.4949) (Table 4).

### Ecological study: models

To test the relative importance of the different factors, two different models were built with the significant factors for each of the *Anopheles* and Culicinae data (Table 5) (see Additional file 1).

**Table 5:** Summary of model parameters and goodness indices

Model parameters			Goodness indices			
Variable type: presence/absence (YN) or count)	Model type	Interaction with season (N = Add/Y = Interact)	Residuals deviance	Null residual	Degrees of freedom	AIC
*Anopheles*						
*YN*	*logreg–binom* (*Ano1*)	*N*	*740.81*	*782.92*	*596*	*762.81*
YN	logreg–binom	Y	710.35	782.92	580	764.35
Count	glmPoisson	N	7437.9	9536.4	596	8189.1
*Count*	*glmPoisson* (*Ano2*)	*Y*	*6831*	*9536.4*	*580*	*7614.1*
*Culex*						
*YN*	*logreg–binom* (*Culex1*)	*N*	*719.22*	*773.65*	*598*	*737.22*
YN	logreg–binom	Y	698.48	773.65	582	748.48
count	glmPoisson	N	22.691	27.036	598	23.528
*count*	*glmPoisson* (*Culex2*)	*Y*	*18,694*	*27,036*	*582*	*19,564*

It shows the residual and Null deviances, degrees of freedom and the Akaike Information Criterion (AIC). The selected models are in italic with names in brackets

For *Anopheles* the two following formulae were used

- *Anopheles* ~ Typology + DistRiver + Up/Down + Water surface, with no interaction between factors
- *Anopheles* ~ (Typology + DistRiver)*Season + Up/Down + Water surface including the Season interaction with both the typology and Distance to the river.

The logistic regression (LR) model improved only slightly the data and the model without interaction with Season gave only by few a better AIC (Table 5) (see Additional file 1). The GLM-Poisson model decreased the number of residuals compared to the null model. The AIC was better with the model with interaction between Season and Typology and Distance to the river. The histogram of residuals showed a skewed distribution (see Additional file 2) and the residual *vs* fit plot show a divergence in the higher values of abundances (see Additional file 3).

The predictor factors ‘Typology’, ‘Distance to the river’, UpDown stream’ were kept in all the models (Table 6). The ‘water surface’ factor was significant only with GLM-Poisson. The predictor factors influenced the models in the same way (Table 6). The ‘Pond’ as typology modality was found significantly and positively associated with the abundance of *Anopheles* (p < 0.0015). A distance higher than 500 m to the river was negatively associated with *Anopheles* abundances (p < 0.035–p < 2e−16) in all models. The ‘up/down stream’ factor gave negative estimates when being at the city or downstream (p < 0.0002), but not significantly in the case of LR. The water surface was negatively associated (less *Anopheles* if greater size of sites) only in GLM model.

**Table 6:** Summary of model results

	Significant variable in the model: Yes/No					Interactions	
	Typology (reference “human made”)	Distance to river (reference “<500 m”)	Up/down stream (reference “upstream”)	Water surface	Season (reference “Dry cool”)	Typology * season	Distance river * season
Ano1	***Kori (p*** ***=*** ***0.0524)***	*Yes (Q*—*p* = *0.0281)*	***(p*** ***=*** ***0.0977)***	No	NA	NA	NA
Ano2	***Pond (p*** ***<*** ***2e−16),*** *River (p* = *0.0217)*	*Yes (Q*—*p* < *2e*−*16)*	*Yes (L*—*p* < *2e*−*16)*	*Yes (p* = *2e*-*6)*	***Rainy (p*** ***=*** ***0.0003)***	Yes	Yes
Culex1	*Pond (p* = *0.0028), River (p* = *0.0027)*	*Yes (L*—*p* = *0.0139)*	NA	No	NA	NA	NA
Culex2	***Kori (p*** ***<*** ***2e−16),*** *Pond (p* < *2e*−*16), Ricefield(p* < *2e*−*16), River (p* = *8.7e*−*11), Small temp(p* = *2e*-*11)*	*Yes (Q*—*p* < *2e*−*16)*	NA	*Yes p* < *2e*−*16*	*Dryhot (p* < *2e*−*16), Rainy (p* < *2e*−*16)*	Yes	***Yes***

Positive relationships are in bolditalic. Negative relationships are italicized. The reference modality in model is indicated in column head. Models are namedafter Table 5. Interactions between factors are detailed in the Table 7

The interaction between factors improved the GLM model, but not the logistic regression model (Table 5). The Rainy season impacted negatively on the abundances of *Anopheles* for ‘Pond’, ‘River’ and ‘Small Temp’ and positively for ‘Ricefield’ (Table 7). The Dry and Hot Season had a negative impact in the cases of ‘Kori’ and ‘Pond’, but a positive impact for the ‘River’. Both the Rainy Season and the Hotand Dry Season had positive interactions with the distance to the river (p < 0.01).

**Table 7:** Significant interactions between factors in GLM models

Typology (reference “Human made”)	*Anopheles*		Culicinae	
	Rainy season	Hot and dry season	Rainy season	Hot and dry season
Kori		Ano2***	Culex2***	
Pond	Ano2***	Ano2***		Culex2***
Ricefield	Ano2**		Culex2***	Culex2***
River		Ano2***	Culex2***	Culex2*
Small Temp	Ano2***		Culex2**	
***Distance river***(L-Linear, Q-Quadratic)	Ano2*** (Q ***+*** ***L)***	Ano2***(L)	Culex2*** (Q)	Culex2*** (Q)

The significant interactions upon mosquito aquatic stages abundances are shown between modalities (classes) of the Season Factor and Typology and Distance to the river. The reference modality is indicated in column or row heads. In bolditalic, positive interactions; italicized, negative interactions; the p values are coded as: 0 ‘***’ 0.001 ‘**’ 0.01 ‘*’ 0.05 ‘·’ 0.1 ‘ ' 1

For the Culicinae, the two following formulae were used:

- Culicinae ~ Typology + DistRiver + Water surface
- Culicinae ~ (Typology + DistRiver)*Season + Water surface, including the Season interaction with both the typology and Distance to the river.

As for *Anopheles*, the LR model improved the data and decreased the residual deviance with also a very small difference between the AIC with or without interactions between variables (Table 5) (see Additional file 1). The GLM Poisson model was better if including the interactions of the Season upon Typology and Distance to the river (Table 5) (see Additional file 1). The histogram of distribution of residuals was better than for *Anopheles* but still deviant from normality (see Additional file 2). As for *Anopheles* the plot of residuals *vs* fitted deviated in the higher values (see Additional file 3). All the predictors were kept: The ‘Pond’ (p < 0.0045) and ‘River’ (p < 0.025) modalities of the Typology are negatively associated with Culicinae in the two models. The ‘Kori’ modality is very significantly and positively associated with the abundances of Culicinae in GLM, but not with their presence in case of LR model (Table 6).

The water surface showed a negative impact in the GLM-Poisson (p < 2e−16). When included in the GLM model, the Season predictor showed a negative impact on Culicinae abundances (p < 2e−16) for both the ‘Rainy and Dry and Hot’ modalities compared to the Dry cool reference modality (Table 7). The interactions between season and the typology classes showed a positive impact of the Rainy Season on the Abundances of Culicinae sampled the ‘RiceField’, ‘Small Temp’ and with the ‘River’ in the GLM-Poisson model (Table 7). The Rainy season had a negative impact on the abundances of Culicinae sampled in the ‘Kori’ for the GLM integrating the interactions. The Hot and Dry Season influenced positively the Culicinae abundances in ‘Ricefield’ and ‘Pond’ and ‘River’. The distance to the river has a positive interaction with the two ‘Rainy’ and ‘Hot and Dry’ seasons (Table 7).

### Adult mosquito study

#### Composition of the population and abundance

Between June 2008 and June 2009, 196 rooms were sprayed with insecticide and 294 light traps set at night giving a total of 11,957 female mosquitoes (Table 8). The total mosquito counts were comparable in the two sites: 6381 for Goudel and 5576 for Boukoki (Fig. 2). Among them, 2139 *Anopheles* females were collected, with 95 % of *An. gambiae s.l.*, 1.7 % *An. rufipes*, 1.7 % *An. ziemanni*, 1.4 % *An. pharoensis* and 0.6 % *An. funestus*. However *An. gambiae s.l.* accounted for 33 % of the mosquitoes captured in the Goudel (peri-urban) area and only 0.7 % in the Boukoki (urban) area. *Anopheles funestus* was found only in the peri-urban area. *Culex* sp. predominated in both districts, accounting for 62 % of the mosquitoes in the peri-urban area and 99 % in the urban area.

**Table 8:** Composition of mosquito fauna

Districts of Niamey								
Species		Goudel (semi-urban area)			Boukoki 4 (urban area)			Total
		PS	LT	Total	PS	LT	Total	
*An. gambiae s.l.*		715	1273	1988	10	27	37	2025
*An. funestus*		2	10	12	0	0	0	12
*An. rufipes*		12	21	33	0	3	3	36
*An. ziemanni*		0	36	36	0	0	0	36
*An. pharoensis*		1	29	30	0	0	0	30
*Culex sp.*		1110	2849	3959	1031	4483	5514	9473
*Mansonia sp.*		1	218	219	1	1	2	221
*Aedes sp.*		1	13	14	0	6	6	20
Phlebotomine		74	16	90	1	13	14	104
		1916	4465	6381	1043	4533	5576	11,957

Composition and counts of female mosquito collected in 98 rooms after morning spraying and in 147 light traps per locality in two districts of Niamey during 1 year (2008–2009); PS means pyrethrum spray and LT means light trap

#### Variation of adult mosquito abundances

**Fig. 6:**
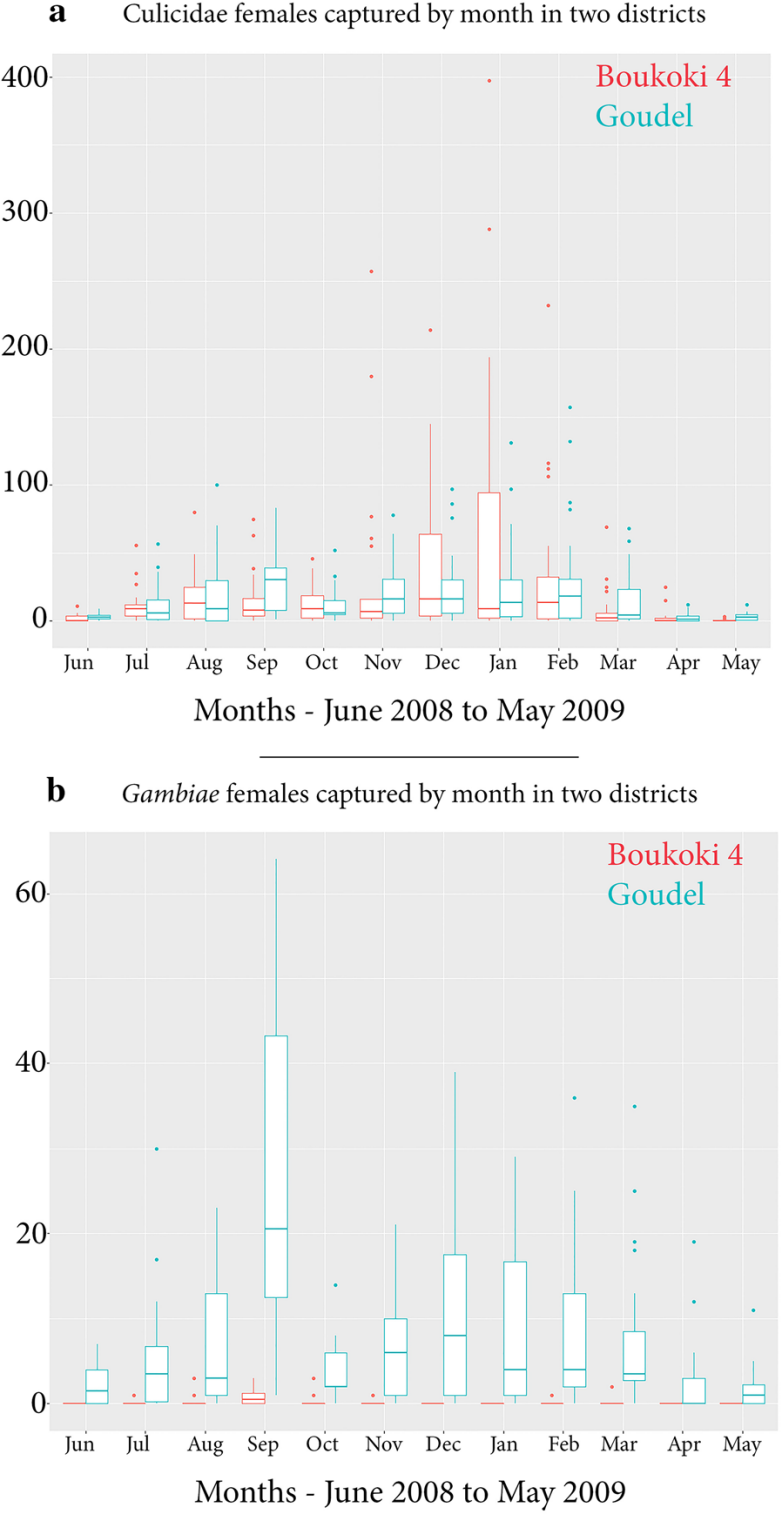
Monthly variation of mosquito abundance. Monthly variation of mosquito abundance showing different temporal dynamics for *Anopheles gambiae* (**b**) compared to the overall Culicidae (**a**) for the peri-urban district close to the river (Goudel) and the urban district close to the kori (Boukoki 4). Note the different y axis scales

 Mean mosquito density per district varied over time (Fig. 6). *An. gambiae s.l.* showed two different seasonal patterns: unimodal, peaking during the rainy season at Boukoki, and bimodal at Goudel, with one high peak during the rainy season, and one wide and smoothed peak during the dry seasons. At Goudel, the maximum capture event for *An. gambiae s.l.* was in September with 64 females in one sprayed room and 59 females in one light trap. The second peak showed a maximum of 39 females for residual spraying in December and36 for light trap, in February. At Boukoki 4, the highest captures of *An. gambiae s.l.* were in August, September and October, peaking only at three females per sampling event. The highest captures of *Culex* mosquitoes were done with light traps from November to February (>150 females per event) in Boukoki, in several occasions. Such a level of *Culex* adult capture was reached only once at Goudel, in February.

#### Circumsporozoite protein detection

**Fig. 7:**
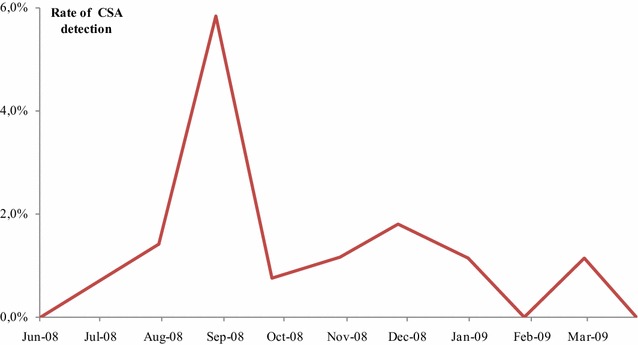
Temporal pattern of change in CSA levels in *An. gambiae s.l.* in Goudel. Temporal pattern of change in CSA levels in *An. gambiae s.l.* in Goudel, a semi-urban area of Niamey

In Boukoki 4 (urban area), none of the 34 females of *An. gambiae s.l.*, tested by ELISA for the presence of the circumsporozoite protein of *P. falciparum* was found positive. In Goudel, nineteen (1.3 %) among 1433 *Anopheles gambiae s.l.* females were found positive by ELISA. CSP-positive *An. gambiae s.l.* Specimens were found from July 2008 up to February 2009 with maximum value in August (Fig. 7).

#### Distribution of *An. gambiae s.l*. complex

**Fig. 8:**
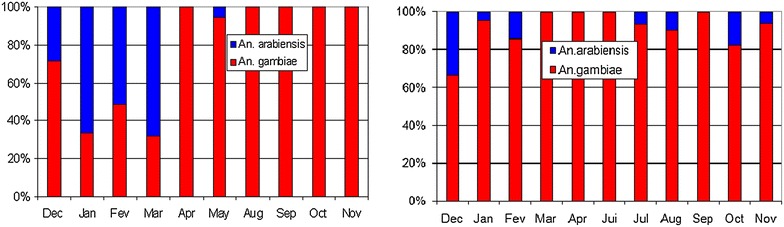
Monthly distribution of *An. gambiae* complex species. Monthly distribution of *An. gambiae* complex species for adults reared from larvae in 2003 (*left*) and for adults collected in 2009 (*right*) from urban areas of Niamey

The distribution by *Anopheles gambiae* complex species of adults caught in pyrethrum spray catches, CDC light-traps and reared from larval collections is shown in Fig. 8. In 2003, 255 out the 2434 *An. gambiae s.l.* emerging adults were genotyped by PCR. The population of adult *An. gambiae* complex mosquitoes bred from larval collections consisted of 56 % *An. gambiae s.s.* (*An. gambiae* + *An.coluzzii*) and 44 % *An. arabiensis.* The relative frequency of the larvae of *An. gambiae s.s.* was particularly high in the rainy season (100 %) and then the cold season, whereas *An. arabiensis* was more frequent in the dry and hotter season (February to May). *Anopheles gambiae s.s.* was found in 34 out of 35 sites whereas *An. arabiensis* was found in only 12 sites. The Goudel pond was the most prevalent site for *An. arabiensis* (79 % of the specimens identified as *An. arabiensis*). Further molecular identification of 103 specimens from 2003 showed 61 % *An. coluzzii* (61 %) and 39 % of *An. gambiae* (=S molecular form). Out of 27 sites, *An. coluzzii* was found in 23 sites whereas *An. gambiae* was found in 11 sites. In 2009, the molecular identification of 431 adult *An. gambiae s.l.* showed 90 % *An. coluzzii* (M form) and 10 % *An. arabiensis,* without any *An. gambiae* (S form) identified. The difference of proportions of *gambiae*/*arabiensis* between 2003 and 2009 is significant (χ^2^ = 105, *p* < 0.0001).

## Discussion

The study aimed at describing and analysing the ecology of urban malaria vectors in a Sahelian environment with high constraints on water sources. The mosquito species that were found during the study are already known from this region [6, 27]. In African urban environments, the most commonly found malaria vectors are: *An. gambiae* and *An. arabiensis* [1]. *Anopheles funestus* has been found in urban environments of Gabon and Mozambique [1], at low abundance in Nigeria [28] and very rarely in Antananarivo (Madagascar) [29]. The case of *An. funestus* is noteworthy, as this species was described reappearing in the Sahel zone and particularly in Niger [30] after being absent since the 1970s [3]. The capture of *An. funestus* during the 2009 collection is the first mention in Niamey city. The absence of *Anopheles funestus* from the aquatic stage survey could be linked to its true absence or to its ability to escape the dipping method. The aquatic net would be probably more appropriate for its collection [31] but this vector was not expected at that time. However, given the vector capacity of this vector, the survey of its potential expansion is of prime importance.

New insights for *An. gambiae* complex distribution in the Sahel are provided. According to genotyping, *An. coluzzii* is the most prevalent species found both in 2003, as aquatic stage, and in 2009, as adult. *An. arabiensis* is found associated with the hot and dry season as in neighbouring rural areas of Niger [6] and confirmed its affinities for arid and dry climate, as in the northern parts of Cameroon [32]. The significant difference of relative proportion of *An*. *arabiensis* and *An. gambiae* between 2003 and 2009 may be related to differences in the sampled population stages (aquatic and adult) but also to changes in the true distribution of these species. Such a change has been observed in rural areas 60 km distant from Niamey [6]. In the same region, the S form (*An. gambiae*) decreased significantly from 2005 to 2007, after a large distribution of impregnated bed nets [6]. In the present study, the disappearance of the S form during the 2009 collection, despite a notable (>400) number of genotyped samples is puzzling and could reveal a general trend for this species. The difference of total rainfall between the two sampling periods is relatively important (−32 % in 2008–2009) but remains within the range of the year-to-year recorded variability within the total period of the study. Therefore, it is difficult to conclude between a short-term, year-to-year, difference or a longer term trend.

The sampling effort and the number of collected pre-imaginal stages are very comparable to a survey led in garden wells of Dakar, Senegal by Robert et al. [33]. However the proportion of *Anopheles* aquatic stages is higher in Niamey than in Dakar (29 vs 12 %). The results show a clear difference in the distribution of aquatic stages by sites and by month (Fig. 3a) between the *Anopheles* and the Culicinae. By different approaches of data analysis, we found environmental factors which seem important for abundances of malaria vectors. The models were not perfectly fit to the aquatic stage collection data. However the Poisson distribution GLM improved the residual deviance by one-third compared to the null deviance. The modelling of the pre-imago malaria vector ecology confirmed the PCA and Kruskall–Wallis test results; it showed the importance of the situation upstream to the city and the importance of the ponds and wells in gardens, as well as the distance to the Niger River. Models allowed to look at the interactions between seasonality and the typology of the breeding sites, as well as the effect of the season on the river, by flooding both in rainy and in the cool and dry seasons, or by the residual breeding sites in the riverbed during the hot and dry season. In Niamey, as in Dakar, *An. gambiae* could be found in relation to garden wells. The Gountou Yena kori is an important site for *Anopheles* breeding. Its situation in continuity with garden drains increases the exposure of *Anopheles* aquatic stages to insecticide. In 2007, the resistant allele frequency of the knock-down resistance gene was found higher in this area than in other areas of the city [34]. In the sub-region, urban agriculture has been shown increasing the abundances of *Anopheles* and *Culex* in Kumasi [35] and Accra [36], Ghana and Dakar, Senegal [33]. In the two last situations, authors discussed not only the role of larval breeding sites but also agriculture sites as potential resting sites for adult mosquitoes.

In a first analysis, the seasonality was not found a significant factor for the distribution of the pre-imago stages of *Anopheles*. Indeed, malaria vectors are found all along the year with different and alternative breeding sites in the zone. In Ouagadougou (Burkina Faso), Fournet et al. [37] also found malaria vectors during the whole year. However, the adult collection conducted in Niamey in 2009 showed a clear temporal signal, particularly in the city centre, with maximal values during the rainy season. This impact was confirmed when modelling the abundance of *Anopheles* larvae and pupae included the interaction of the season with the typology of breeding sites. The impact of the season could be different according to the types of breeding sites: a positive impact of the rainy season in the rice field and a negative for the breeding sites associated with the river and the small and temporary sites. The influence of the rains flushing out the small breeding sites, included the ones created in the river bed by the previous dry season could be locally important. The ‘Pond’ type is negatively associated by both the ‘rainy’ and the ‘hot and dry’ seasons (Table 7) for influencing the *Anopheles* aquatic stage abundances. Indeed, these types of breeding sites will favour higher abundances of *Anopheles* during the dry and cold seasons, after the rainy season, as shown by the adults dynamics near the Goudel pond: here, the abundance temporal peak of *An. gambiae* adults is much wider (Fig. 6) than in the city centre and widens the season of abundance up to the drier seasons. The sporozoite index estimated in the Goudel zone, near the pond is 1.3 % and is close to the index found in Zindarou at 1.4 % in 2007, after the insecticide impregnated bed net distribution [6]. No infected *Anopheles* was found in Boukoki in the city centre. As the number of sites for adult collection is limited, we cannot extrapolate for the whole city. However we have shown that notable malaria transmission still occurs in the peri-urban area. The Goudel area is at high risk for malaria and after local health centre data, people do experience the malaria burden. In the centre of the city, the Gountou Yena kori is also a hot spot for malaria vectors.

The city has changed during the 7 years separating the beginning and the end of the study. However, most of the changes in urban structure have occurred in the Northern and Eastern boundaries of the city, far from the heart of the city and the river, which was the main focus of the study. The ecological factors identified here are not outdated and provide important features of the ecology of malaria vectors in Niamey. These data represent the first steps for the potential larval source management [38] as control method. The Goudel Pond, and the Gountou Yena kori and its proximity are identified as permanent or semi-permanent sources of *Anopheles*. If models could be considered as relatively accurate, the proximity of the river is confirmed as a risk factor for *Anopheles*. The upstream location of the breeding sites is a newly identified risk factor for *Anopheles* in Niamey. However many sites are transitory and may make larval control difficult [36]. However the quantification of the parasite transmission by the measure of sporozoite index within the city is an important step forward. In complement to the LLINs strategy already implemented, stakeholders should ask if the larval source management could be a cost-effective approach [10] to improve the malaria control for the community of Niamey.

## Conclusion

Malaria transmission is occurring in Niamey. In the peripheral area, the sporozoite index is comparable to local rural villages. However, aquatic stages are not distributed randomly and distribution factors have been identified. Encouraged by the Dar-es-Salaam experience [12], the results of the study show that the vector malaria control could focus on key-areas and provide seeds for future works on larval and pupae ecology in Niamey.

## Additional files

10.1186/s12936-016-1352-0 Model parameters and main results. Models using *Anopheles* data and *Culex* data are detailed with formulas and main results.
10.1186/s12936-016-1352-0 Residuals histograms. Residuals histogram with normal distribution curve for 4 models.
10.1186/s12936-016-1352-0 Residuals *vs* fit plots. Plots of the residuals against the fitted values side by side for GLM Poisson models for *Anopheles* and Culicinae counts.
