# Supplementary material for: Ecology of urban malaria vectors in Niamey, Republic of Niger
Source: Malar J. 2016 Jun 8;15:314. doi: 10.1186/s12936-016-1352-0 (PMC4898306; doi:10.1186/s12936-016-1352-0)
Supplement: Supplementary file 1 — 10.1186/s12936-016-1352-0 Model parameters and main results. Models using Anopheles data and Culex data are detailed with formulas and main results. [file 12936_2016_1352_MOESM1_ESM.docx]

Models parameters and main results

- The different models are highlighted in yellow.
- Codes of the model names are: Ano=”Anopheles”, Cul= “Culex”, Count= collected mosquitoes as discrete quantitative variable, YN = collected mosquitoes as presence/absence variable, Add= simple addtive model, Interact= model with interactions between Season X Typology (ClassGeneral) and Season X distance to the river (DistFlFAC), Pois= “Poisson family”, binom= “binomial family”, glm=generalized linear model, log reg= logistic regression (for presence / absence) which is equivalent to binomial family glm.
- Indices of goodness are highlited in green.
- Significant model variables are in blue for selected models.

# *Anopheles*

## LogReg

### glmbinomAnoYNAdd

glm(formula = AnoYN ~ ClassGeneral + DistFlFAC + UpDownFAC + watsurf, family = binomial(logit), data = NiameyNoZero)

Deviance Residuals:

Min 1Q Median 3Q Max

-1.5511 -0.9312 -0.6824 1.2346 1.8107

Coefficients:

Estimate Std. Error z value Pr(>|z|)

(Intercept) -0.51913 0.29042 -1.788 0.0739 .

ClassGeneral[T.Kori] 1.42364 0.73397 1.940 0.0524 .

ClassGeneral[T.Pond] -0.06069 0.32770 -0.185 0.8531

ClassGeneral[T.Ricefield] 0.32914 0.29467 1.117 0.2640

ClassGeneral[T.River] -0.22896 0.29590 -0.774 0.4391

ClassGeneral[T.SmallTemp] 1.01315 0.74305 1.364 0.1727

DistFlFAC.L 0.40616 0.21609 1.880 0.0602 .

DistFlFAC.Q -0.42438 0.19330 -2.195 0.0281 *

UpDownFAC.L -0.22649 0.19008 -1.192 0.2334

UpDownFAC.Q 0.28060 0.16943 1.656 0.0977 .

watsurf 0.01414 0.06049 0.234 0.8152

---

Signif. codes: 0 '***' 0.001 '**' 0.01 '*' 0.05 '.' 0.1 ' ' 1

(Dispersion parameter for binomial family taken to be 1)

Null deviance: 782.92 on 606 degrees of freedom

Residual deviance: 740.81 on 596 degrees of freedom

AIC: 762.81

### glmbinomAnoYNInteract

glm(formula = AnoYN ~ (ClassGeneral + DistFlFAC) * Season + UpDownFAC + watsurf, family = binomial(logit), data = NiameyNoZero)

Deviance Residuals:

Min 1Q Median 3Q Max

-1.4682 -0.8861 -0.6592 1.1791 1.9438

Coefficients:

Estimate Std. Error z value Pr(>|z|)

(Intercept) -2.703e-01 4.147e-01 -0.652 0.514572

ClassGeneral[T.Kori] 1.302e+00 1.495e+00 0.871 0.383588

ClassGeneral[T.Pond] -6.283e-02 5.924e-01 -0.106 0.915537

ClassGeneral[T.Ricefield] -2.869e-01 5.147e-01 -0.557 0.577188

ClassGeneral[T.River] -1.155e+00 5.062e-01 -2.282 0.022487 *

ClassGeneral[T.SmallTemp] 1.538e+01 7.277e+02 0.021 0.983141

DistFlFAC.L -7.224e-01 4.096e-01 -1.763 0.077821 .

DistFlFAC.Q -7.463e-01 3.633e-01 -2.054 0.039967 *

Season[T.DryHot] -2.495e-01 4.508e-01 -0.553 0.579949

Season[T.Rainy] -4.444e-01 4.169e-01 -1.066 0.286495

UpDownFAC.L -1.699e-01 1.958e-01 -0.868 0.385552

UpDownFAC.Q 2.982e-01 1.733e-01 1.721 0.085306 .

watsurf 6.691e-03 6.429e-02 0.104 0.917108

ClassGeneral[T.Kori]:Season[T.DryHot] -1.331e+00 1.962e+00 -0.678 0.497727

ClassGeneral[T.Pond]:Season[T.DryHot] 1.977e-01 8.697e-01 0.227 0.820174

ClassGeneral[T.Ricefield]:Season[T.DryHot] 6.321e-02 8.067e-01 0.078 0.937539

ClassGeneral[T.River]:Season[T.DryHot] 9.156e-01 7.786e-01 1.176 0.239615

ClassGeneral[T.SmallTemp]:Season[T.DryHot] -2.997e+01 1.627e+03 -0.018 0.985305

ClassGeneral[T.Kori]:Season[T.Rainy] 1.452e+01 6.509e+02 0.022 0.982198

ClassGeneral[T.Pond]:Season[T.Rainy] 6.805e-02 7.487e-01 0.091 0.927578

ClassGeneral[T.Ricefield]:Season[T.Rainy] 1.256e+00 6.727e-01 1.868 0.061800 .

ClassGeneral[T.River]:Season[T.Rainy] 1.710e+00 6.676e-01 2.561 0.010427 *

ClassGeneral[T.SmallTemp]:Season[T.Rainy] -1.500e+01 7.277e+02 -0.021 0.983553

DistFlFAC.L:Season[T.DryHot] 1.280e+00 5.782e-01 2.213 0.026913 *

DistFlFAC.Q:Season[T.DryHot] -3.829e-02 5.638e-01 -0.068 0.945849

DistFlFAC.L:Season[T.Rainy] 1.752e+00 5.215e-01 3.359 0.000781 ***

DistFlFAC.Q:Season[T.Rainy] 6.016e-01 4.598e-01 1.308 0.190734

---

Signif. codes: 0 '***' 0.001 '**' 0.01 '*' 0.05 '.' 0.1 ' ' 1

(Dispersion parameter for binomial family taken to be 1)

Null deviance: 782.92 on 606 degrees of freedom

Residual deviance: 710.35 on 580 degrees of freedom

AIC: 764.35

## GLM

### glmPoisAnocountAdd

glm(formula = AbsAno ~ ClassGeneral + DistFlFAC + UpDownFAC + watsurf, family = poisson(log), data = NiameyNoZero)

Deviance Residuals:

Min 1Q Median 3Q Max

-7.6281 -2.4247 -1.7269 -0.6912 22.1088

Coefficients:

Estimate Std. Error z value Pr(>|z|)

(Intercept) 1.80449 0.07121 25.340 < 2e-16 ***

ClassGeneral[T.Kori] 0.69163 0.10240 6.754 1.43e-11 ***

ClassGeneral[T.Pond] 0.94868 0.06672 14.219 < 2e-16 ***

ClassGeneral[T.Ricefield] 0.26522 0.08042 3.298 0.000974 ***

ClassGeneral[T.River] -0.25630 0.08020 -3.196 0.001394 **

ClassGeneral[T.SmallTemp] -0.73224 0.23133 -3.165 0.001549 **

DistFlFAC.L 0.75347 0.05326 14.147 < 2e-16 ***

DistFlFAC.Q -0.33771 0.04069 -8.300 < 2e-16 ***

UpDownFAC.L -1.15014 0.04469 -25.739 < 2e-16 ***

UpDownFAC.Q -0.12612 0.04097 -3.078 0.002085 **

watsurf -0.10504 0.01562 -6.723 1.78e-11 ***

---

Signif. codes: 0 '***' 0.001 '**' 0.01 '*' 0.05 '.' 0.1 ' ' 1

(Dispersion parameter for poisson family taken to be 1)

Null deviance: 9536.4 on 606 degrees of freedom

Residual deviance: 7437.9 on 596 degrees of freedom

AIC: 8189.1

### glmpoisAnocountInteract

glm(formula = AbsAno ~ (ClassGeneral + DistFlFAC) * Season + UpDownFAC + watsurf, family = poisson(log), data = NiameyNoZero)

Deviance Residuals:

Min 1Q Median 3Q Max

-7.8156 -2.3043 -1.6704 -0.6002 19.9553

Coefficients:

Estimate Std. Error z value Pr(>|z|)

(Intercept) 1.21579 0.12725 9.554 < 2e-16 ***

ClassGeneral[T.Kori] 0.59953 0.48341 1.240 0.214896

ClassGeneral[T.Pond] 1.48474 0.13030 11.395 < 2e-16 ***

ClassGeneral[T.Ricefield] 0.06526 0.14963 0.436 0.662745

ClassGeneral[T.River] -0.34217 0.14910 -2.295 0.021740 *

ClassGeneral[T.SmallTemp] -0.11035 0.26245 -0.420 0.674145

DistFlFAC.L -0.42436 0.13890 -3.055 0.002249 **

DistFlFAC.Q -0.89216 0.09086 -9.819 < 2e-16 ***

Season[T.DryHot] 0.27419 0.14257 1.923 0.054462 .

Season[T.Rainy] 0.45919 0.12781 3.593 0.000327 ***

UpDownFAC.L -1.05734 0.04668 -22.649 < 2e-16 ***

UpDownFAC.Q -0.10719 0.04099 -2.615 0.008918 **

watsurf -0.08057 0.01706 -4.723 2.32e-06 ***

ClassGeneral[T.Kori]:Season[T.DryHot] -2.41041 0.69831 -3.452 0.000557 ***

ClassGeneral[T.Pond]:Season[T.DryHot] -0.65561 0.16211 -4.044 5.25e-05 ***

ClassGeneral[T.Ricefield]:Season[T.DryHot] -0.16326 0.23061 -0.708 0.478986

ClassGeneral[T.River]:Season[T.DryHot] 1.20784 0.29493 4.095 4.21e-05 ***

ClassGeneral[T.SmallTemp]:Season[T.DryHot] -11.35676 172.65485 -0.066 0.947555

ClassGeneral[T.Kori]:Season[T.Rainy] 0.48824 0.49417 0.988 0.323155

ClassGeneral[T.Pond]:Season[T.Rainy] -0.90972 0.16409 -5.544 2.96e-08 ***

ClassGeneral[T.Ricefield]:Season[T.Rainy] 0.58386 0.18494 3.157 0.001594 **

ClassGeneral[T.River]:Season[T.Rainy] 0.17462 0.18942 0.922 0.356584

ClassGeneral[T.SmallTemp]:Season[T.Rainy] -2.68023 1.03712 -2.584 0.009757 **

DistFlFAC.L:Season[T.DryHot] 2.26523 0.21346 10.612 < 2e-16 ***

DistFlFAC.Q:Season[T.DryHot] -0.29818 0.14084 -2.117 0.034243 *

DistFlFAC.L:Season[T.Rainy] 1.30475 0.15709 8.306 < 2e-16 ***

DistFlFAC.Q:Season[T.Rainy] 1.05692 0.11281 9.369 < 2e-16 ***

---

Signif. codes: 0 '***' 0.001 '**' 0.01 '*' 0.05 '.' 0.1 ' ' 1

(Dispersion parameter for poisson family taken to be 1)

Null deviance: 9536.4 on 606 degrees of freedom

Residual deviance: 6831.0 on 580 degrees of freedom

AIC: 7614.1

# *Culex*

## LogReg

### glmBinomCulYNAdd

glm(formula = CulYN ~ ClassGeneral + DistFlFAC + watsurf, family = binomial(logit), data = NiameyNoZero)

Deviance Residuals:

Min 1Q Median 3Q Max

-1.3533 -0.8654 -0.6466 1.1407 1.9016

Coefficients:

Estimate Std. Error z value Pr(>|z|)

(Intercept) -0.164174 0.280637 -0.585 0.55854

ClassGeneral[T.Kori] 0.308570 0.676592 0.456 0.64834

ClassGeneral[T.Pond] -1.011870 0.338812 -2.987 0.00282 **

ClassGeneral[T.Ricefield] -0.247605 0.283144 -0.874 0.38185

ClassGeneral[T.River] -0.877914 0.292650 -3.000 0.00270 **

ClassGeneral[T.SmallTemp] 0.849293 0.716295 1.186 0.23575

DistFlFAC.L 0.497889 0.202429 2.460 0.01391 *

DistFlFAC.Q -0.313357 0.196231 -1.597 0.11029

watsurf 0.009009 0.062087 0.145 0.88462

---

Signif. codes: 0 '***' 0.001 '**' 0.01 '*' 0.05 '.' 0.1 ' ' 1

(Dispersion parameter for binomial family taken to be 1)

Null deviance: 773.65 on 606 degrees of freedom

Residual deviance: 719.22 on 598 degrees of freedom

AIC: 737.22

### glmbinomCulYNInteract

glm(formula = CulYN ~ (ClassGeneral + DistFlFAC) * Season + watsurf, family = binomial(probit), data = NiameyNoZero)

Deviance Residuals:

Min 1Q Median 3Q Max

-1.7975 -0.8826 -0.6488 1.1467 2.1243

Coefficients:

Estimate Std. Error z value Pr(>|z|)

(Intercept) 0.01438 0.24370 0.059 0.9529

ClassGeneral[T.Kori] 5.16200 166.19410 0.031 0.9752

ClassGeneral[T.Pond] -0.32297 0.35578 -0.908 0.3640

ClassGeneral[T.Ricefield] -0.28109 0.31846 -0.883 0.3774

ClassGeneral[T.River] -0.61878 0.30870 -2.004 0.0450 *

ClassGeneral[T.SmallTemp] 0.86692 0.73364 1.182 0.2373

DistFlFAC.L 0.05568 0.23383 0.238 0.8118

DistFlFAC.Q -0.35908 0.21926 -1.638 0.1015

Season[T.DryHot] -0.15567 0.26526 -0.587 0.5573

Season[T.Rainy] -0.02018 0.24309 -0.083 0.9338

watsurf -0.01261 0.03842 -0.328 0.7427

ClassGeneral[T.Kori]:Season[T.DryHot] -9.93101 214.55423 -0.046 0.9631

ClassGeneral[T.Pond]:Season[T.DryHot] -0.53968 0.58328 -0.925 0.3548

ClassGeneral[T.Ricefield]:Season[T.DryHot] 0.26804 0.47479 0.565 0.5724

ClassGeneral[T.River]:Season[T.DryHot] 0.02192 0.44848 0.049 0.9610

ClassGeneral[T.SmallTemp]:Season[T.DryHot] -5.49323 235.03496 -0.023 0.9814

ClassGeneral[T.Kori]:Season[T.Rainy] -4.68447 166.19543 -0.028 0.9775

ClassGeneral[T.Pond]:Season[T.Rainy] -0.35691 0.46095 -0.774 0.4388

ClassGeneral[T.Ricefield]:Season[T.Rainy] 0.21857 0.40559 0.539 0.5900

ClassGeneral[T.River]:Season[T.Rainy] 0.25498 0.39394 0.647 0.5175

ClassGeneral[T.SmallTemp]:Season[T.Rainy] -0.41909 0.98308 -0.426 0.6699

DistFlFAC.L:Season[T.DryHot] 0.05111 0.33474 0.153 0.8787

DistFlFAC.Q:Season[T.DryHot] 0.02159 0.34425 0.063 0.9500

DistFlFAC.L:Season[T.Rainy] 0.53931 0.30147 1.789 0.0736 .

DistFlFAC.Q:Season[T.Rainy] 0.36865 0.28181 1.308 0.1908

---

Signif. codes: 0 '***' 0.001 '**' 0.01 '*' 0.05 '.' 0.1 ' ' 1

(Dispersion parameter for binomial family taken to be 1)

Null deviance: 773.65 on 606 degrees of freedom

Residual deviance: 698.48 on 582 degrees of freedom

AIC: 748.48

## GLM

### glmPoisCulCountAdd

glm(formula = AbsCul ~ ClassGeneral + DistFlFAC + watsurf, family = poisson(log), data = NiameyNoZero)

Deviance Residuals:

Min 1Q Median 3Q Max

-13.804 -4.136 -2.760 -1.963 44.728

Coefficients:

Estimate Std. Error z value Pr(>|z|)

(Intercept) 3.51594 0.04142 84.882 <2e-16 ***

ClassGeneral[T.Kori] 1.87289 0.04583 40.868 <2e-16 ***

ClassGeneral[T.Pond] -1.53261 0.06985 -21.940 <2e-16 ***

ClassGeneral[T.Ricefield] -1.16437 0.04548 -25.603 <2e-16 ***

ClassGeneral[T.River] -0.67148 0.04218 -15.920 <2e-16 ***

ClassGeneral[T.SmallTemp] -1.29388 0.15174 -8.527 <2e-16 ***

DistFlFAC.L -0.02944 0.02893 -1.018 0.309

DistFlFAC.Q -0.67079 0.02764 -24.273 <2e-16 ***

watsurf -0.17913 0.01019 -17.584 <2e-16 ***

---

Signif. codes: 0 '***' 0.001 '**' 0.01 '*' 0.05 '.' 0.1 ' ' 1

(Dispersion parameter for poisson family taken to be 1)

Null deviance: 27036 on 606 degrees of freedom

Residual deviance: 22691 on 598 degrees of freedom

AIC: 23528

### glmPoisCulCountInteract

glm(formula = AbsCul ~ (ClassGeneral + DistFlFAC) * Season + watsurf, family = poisson(log), data = NiameyNoZero)

Deviance Residuals:

Min 1Q Median 3Q Max

-25.052 -3.861 -2.762 -1.381 35.062

Coefficients:

Estimate Std. Error z value Pr(>|z|)

(Intercept) 3.943432 0.050089 78.728 < 2e-16 ***

ClassGeneral[T.Kori] 3.410138 0.067548 50.485 < 2e-16 ***

ClassGeneral[T.Pond] -2.161136 0.104412 -20.698 < 2e-16 ***

ClassGeneral[T.Ricefield] -2.330717 0.082539 -28.238 < 2e-16 ***

ClassGeneral[T.River] -0.916865 0.065455 -14.008 < 2e-16 ***

ClassGeneral[T.SmallTemp] -1.714713 0.256910 -6.674 2.48e-11 ***

DistFlFAC.L -0.121038 0.054934 -2.203 0.02757 *

DistFlFAC.Q -1.608263 0.039930 -40.277 < 2e-16 ***

Season[T.DryHot] -1.172075 0.056078 -20.901 < 2e-16 ***

Season[T.Rainy] -0.741912 0.045169 -16.425 < 2e-16 ***

watsurf -0.167482 0.010706 -15.643 < 2e-16 ***

ClassGeneral[T.Kori]:Season[T.DryHot] -16.629548 99.638918 -0.167 0.86745

ClassGeneral[T.Pond]:Season[T.DryHot] 2.050825 0.153931 13.323 < 2e-16 ***

ClassGeneral[T.Ricefield]:Season[T.DryHot] 2.139467 0.124233 17.221 < 2e-16 ***

ClassGeneral[T.River]:Season[T.DryHot] 0.202789 0.102681 1.975 0.04828 *

ClassGeneral[T.SmallTemp]:Season[T.DryHot] -12.280384 172.654709 -0.071 0.94330

ClassGeneral[T.Kori]:Season[T.Rainy] -4.508188 0.214105 -21.056 < 2e-16 ***

ClassGeneral[T.Pond]:Season[T.Rainy] -0.001782 0.201724 -0.009 0.99295

ClassGeneral[T.Ricefield]:Season[T.Rainy] 1.786458 0.109329 16.340 < 2e-16 ***

ClassGeneral[T.River]:Season[T.Rainy] 0.384881 0.093277 4.126 3.69e-05 ***

ClassGeneral[T.SmallTemp]:Season[T.Rainy] 1.007524 0.318373 3.165 0.00155 **

DistFlFAC.L:Season[T.DryHot] -0.350134 0.080750 -4.336 1.45e-05 ***

DistFlFAC.Q:Season[T.DryHot] 1.829775 0.086317 21.198 < 2e-16 ***

DistFlFAC.L:Season[T.Rainy] 0.402457 0.070555 5.704 1.17e-08 ***

DistFlFAC.Q:Season[T.Rainy] 1.707637 0.064831 26.340 < 2e-16 ***

---

Signif. codes: 0 '***' 0.001 '**' 0.01 '*' 0.05 '.' 0.1 ' ' 1

(Dispersion parameter for poisson family taken to be 1)

Null deviance: 27036 on 606 degrees of freedom

Residual deviance: 18694 on 582 degrees of freedom

AIC: 19564
