# Supplementary figures and images for: Ecology of urban malaria vectors in Niamey, Republic of Niger
Source: Malar J. 2016 Jun 8;15:314. doi: 10.1186/s12936-016-1352-0 (PMC4898306; doi:10.1186/s12936-016-1352-0)

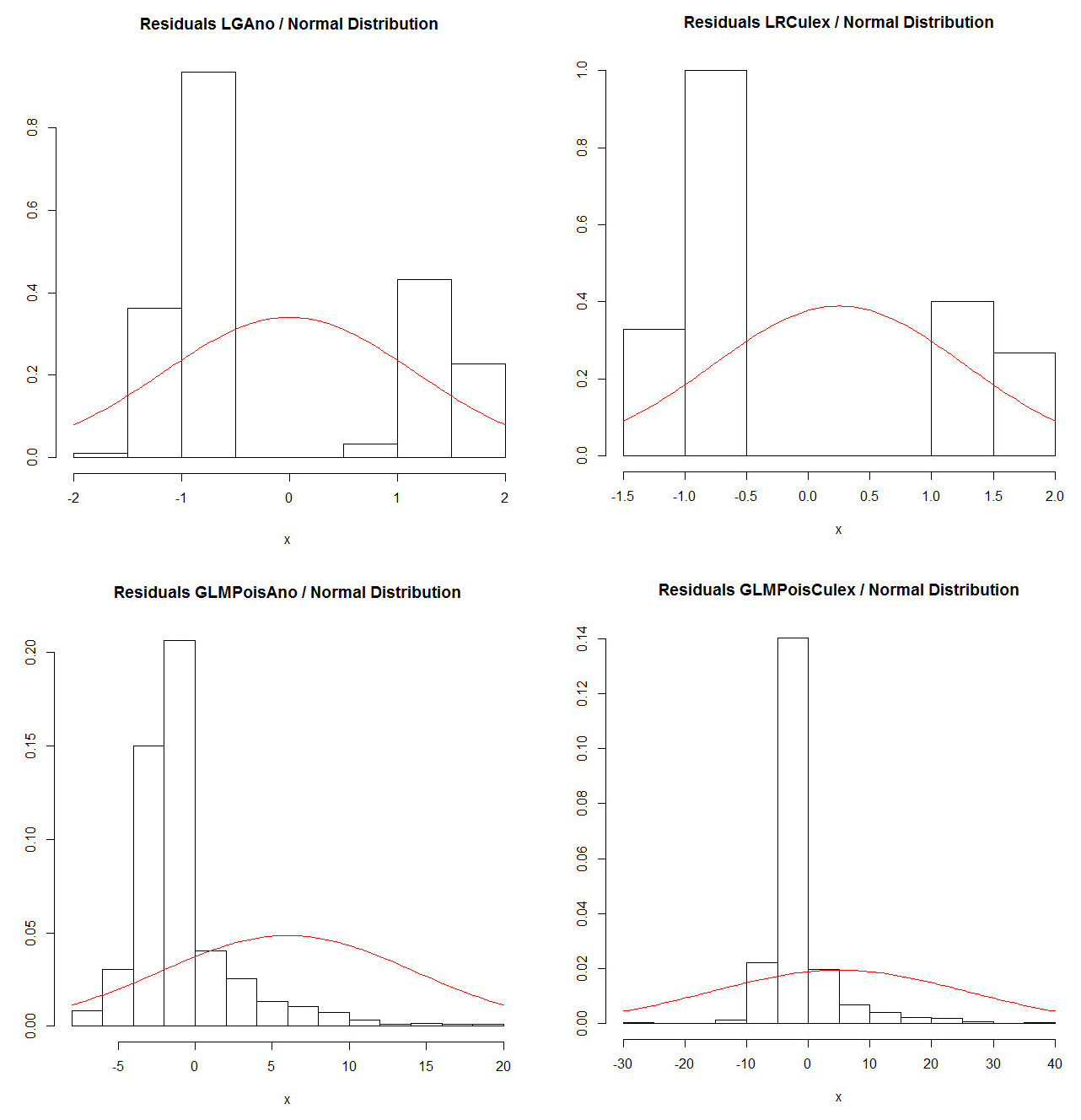

Supplement: Supplementary file 2 — 10.1186/s12936-016-1352-0 Residuals histograms. Residuals histogram with normal distribution curve for 4 models. [file 12936_2016_1352_MOESM2_ESM.jpg]

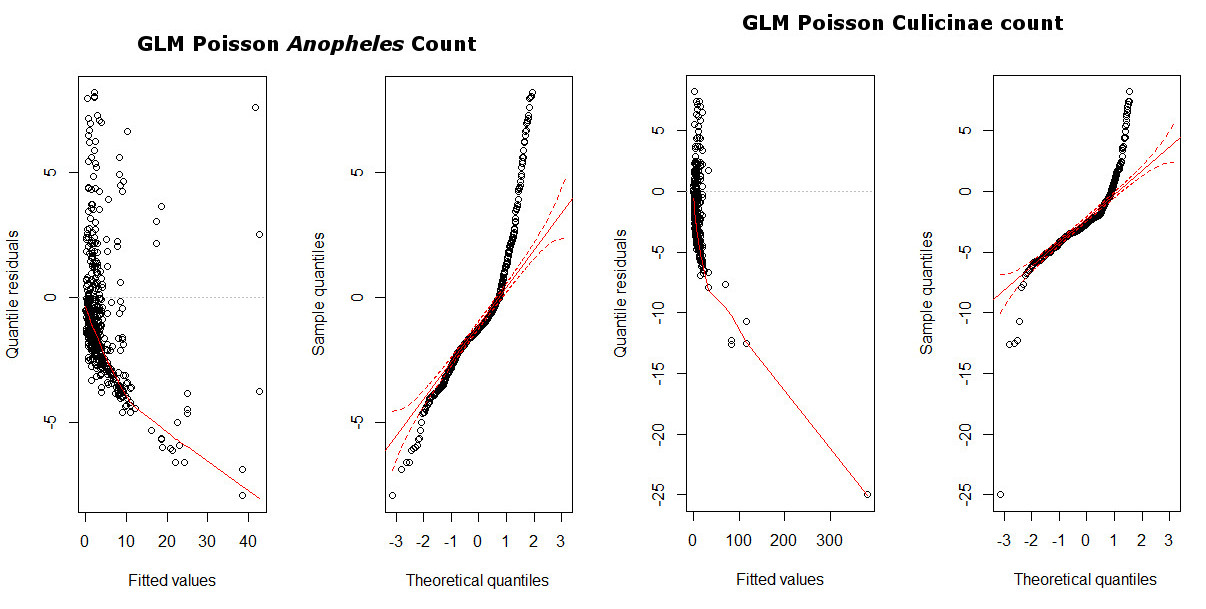

Supplement: Supplementary file 3 — 10.1186/s12936-016-1352-0 Residuals vs fit plots. Plots of the residuals against the fitted values side by side for GLM Poisson models for Anopheles and Culicinae counts. [file 12936_2016_1352_MOESM3_ESM.jpg]
